# Spontaneous formation and base pairing of plausible prebiotic nucleotides in water

**DOI:** 10.1038/ncomms11328

**Published:** 2016-04-25

**Authors:** Brian J. Cafferty, David M. Fialho, Jaheda Khanam, Ramanarayanan Krishnamurthy, Nicholas V. Hud

**Affiliations:** 1 School of Chemistry and Biochemistry, Georgia Institute of Technology, 901 Atlantic Drive, Atlanta, Georgia 30332, USA; 2 NSF-NASA Center for Chemical Evolution, Atlanta, Georgia 30332, USA; 3 Department of Chemistry, The Scripps Research Institute, La Jolla, California 92037, USA

## Abstract

The RNA World hypothesis presupposes that abiotic reactions originally produced nucleotides, the monomers of RNA and universal constituents of metabolism. However, compatible prebiotic reactions for the synthesis of complementary (that is, base pairing) nucleotides and mechanisms for their mutual selection within a complex chemical environment have not been reported. Here we show that two plausible prebiotic heterocycles, melamine and barbituric acid, form glycosidic linkages with ribose and ribose-5-phosphate in water to produce nucleosides and nucleotides in good yields. Even without purification, these nucleotides base pair in aqueous solution to create linear supramolecular assemblies containing thousands of ordered nucleotides. Nucleotide anomerization and supramolecular assemblies favour the biologically relevant β-anomer form of these ribonucleotides, revealing abiotic mechanisms by which nucleotide structure and configuration could have been originally favoured. These findings indicate that nucleotide formation and selection may have been robust processes on the prebiotic Earth, if other nucleobases preceded those of extant life.

## Abstract (web summary)

One of the questions for prebiotic chemistry is the formation of complementary base pairing systems. Here, the authors show that plausible two prebiotic heterocycles can form glycosidic bonds with ribose in water and that these spontaneously base-pair in aqueous solution.

## Graphical abstract

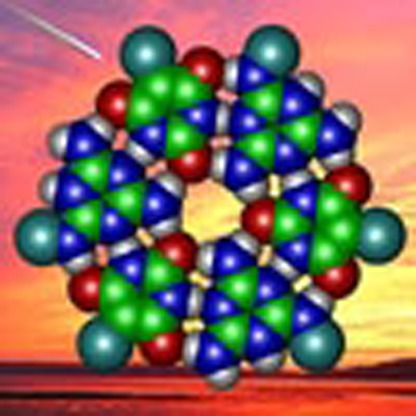

The current role of mononucleotides and RNA polymers in numerous cellular functions gave rise to the long-standing hypothesis that these molecules were involved early in the emergence of life: the RNA World hypothesis^1^. Supporting this hypothesis, model prebiotic reactions and analyses of carbonaceous meteorites provide evidence that the canonical nucleobases of RNA (adenine, guanine, cytosine, uracil) were likely present on the prebiotic Earth^2^,^3^,^4^. In addition, progress has been made towards finding abiotic routes to ribose and related sugars from simple molecules (for example, formaldehyde, glyoxylate), as well as mechanisms for ribose phosphorylation and ribose selection from complex sugar mixtures^5^,^6^,^7^,^8^. Nevertheless, despite decades of effort, the chemical origin of nucleosides and nucleotides (that is, nucleobases glycosylated with ribose and phosphorylated ribose) remains an unsolved problem^9^,^10^. In the 1970s, Orgel and co-workers showed that adenosine (the nucleoside of adenine) can be formed in 1–5% yield if a solution of adenine and ribose is dried and heated^11^,^12^, but no comparable reactions have been demonstrated for the other three canonical nucleosides. Frustrated by what became known as *The Nucleoside Problem*, Orgel proposed an alternative pathway for prebiotic pyrimidine nucleoside and nucleotide synthesis that bypassed glycosidic bond formation, a pathway in which the cytosine nucleobase is built stepwise on a sugar scaffold^13^. This approach was furthered by Sutherland and co-workers, who developed a synthetic route to cytidine and uridine nucleotides starting from glycolaldehyde and glyceraldehyde^13^,^14^. However, the necessity to temporally separate specific reagents and reaction steps caused some to question the relevance of this synthesis to the origin of RNA^15^,^16^. Sutherland and co-workers have since proposed a spatially separated geochemical scenario that includes an ordered delivery of reagents on the prebiotic Earth that would coincide with the sequential steps of their pyrimidine nucleotide synthesis, a scenario that is initiated by meteorite impacts^17^.

The persistent challenge of finding a simple, robust and plausible prebiotic route to the canonical nucleosides—juxtaposed with the exquisite functionality of RNA—have caused many researches to consider RNA a product of chemical and/or biological evolution^18^,^19^. Inspired by the possibility that RNA evolved from a *proto*-RNA with alternative nucleobases that more easily formed nucleosides, Miller and co-workers demonstrated that urazole (a triazole analog of uracil) is efficiently glycosylated by ribose in water^20^. Subsequent demonstrations of nucleoside formation with different plausible prebiotic heterocycles^12^,^21^,^22^ suggest that other nucleosides may have been common on the prebiotic Earth. While encouraging, a model prebiotic reaction has yet to be reported that produces two extant nucleosides that form a Watson–Crick base pair or two noncanonical nucleosides that form a similar base pair—a property used by extant life for information transfer and, arguably, essential for the emergence of RNA-based life.

As part of our search for the possible components of proto-RNA, we evaluated a series of heterocycles for the ability to be spontaneously glycosylated by ribose and to selectively assemble in water^23^,^24^. In this work, we report the glycosylation reactions and nucleotide assembly/mutual selection of two candidate proto-nucleobases, barbituric acid (BA) and melamine. These heterocycles are well suited to function as the recognition units of a primitive informational system, as BA and melamine: (i) are produced in the same model prebiotic reaction^25^; (ii) can form Watson–Crick-like base pairs with each other^26^; (iii) have H-bond donor and acceptor groups that are complementary with uracil (for melamine) and adenine (for BA), making these heterocycles ‘forward compatible' for base pairing with two extant nucleobases^9^; (iv) possess chemical properties that indicate favourable glycosylation by ribose, specifically at the C5 position of BA and the exocyclic amines of melamine. Additional reasons have been previously put forth for why BA and melamine should be considered potential ancestral nucleobases of RNA^27^,^28^.

Here, we show that glycosylation of melamine and BA by ribose-5-phosphate (R5P) occurs spontaneously in water to produce nucleotides in yields of up to 55% and 82%, respectively. When combined, the nucleotides form supramolecular assemblies with Watson–Crick-like base pairs, even within the crude reaction mixtures. These assemblies are shown to preferentially incorporate and increase the fraction of the β-anomer of the melamine nucleotide over the α-anomer. These findings demonstrate prebiotically plausible mechanisms for the selection of nucleotides in both nucleobase and sugar structure.

## Results

### Spontaneous formation of nucleosides and nucleotides

**Figure 1:**
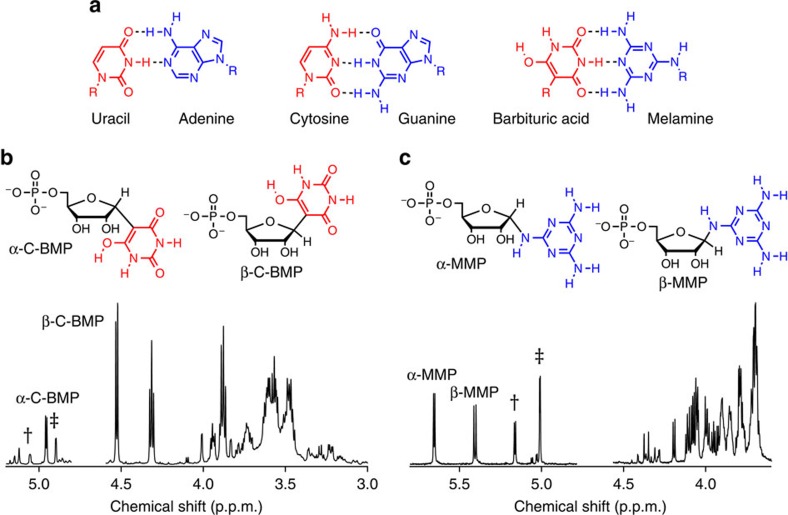
Spontaneous formation of nucleotides by barbituric acid (BA) and melamine in water. (**a**) Chemical structures of the four canonical nucleobases of RNA shown in their Watson–Crick base pairs, and BA with melamine in an analogous Watson–Crick-like base pair. The R group on all nucleobases, which is H for the free nucleobases, indicates the position of ribose attachment through a glycosidic bond on the canonical bases and for melamine and BA in the current work. (**b**) Chemical structures of the two C-nucleotide anomers of BA-ribosyl-monophosphate (C-BMP) and ^1^H NMR spectrum of a BA+R5P crude reaction mixture revealing the formation of α-C-BMP and β-C-BMP. (**c**) Chemical structures of the two anomers of melamine-ribosyl-monophosphate (MMP) and the ^1^H NMR spectrum of a melamine+R5P crude reaction mixture revealing the formation of α-MMP and β-MMP. The anomeric proton resonances for each nucleotide are labelled, and those for R5P are marked with † indicating α-R5P, and ‡ indicating β-R5P. Relative integrated intensities of the nucleotide anomeric resonances show that, for these two reactions, the total C-BMP yield was 82%, and the total MMP yield was 55%. The HOD peaks have been removed from the NMR spectra for clarity. See the Methods for reaction details.

Given previous reports that phosphorylated sugars can be produced in model prebiotic reactions^7^, we were motivated to explore the potential for BA and melamine to be glycosylated by R5P, which could represent a model prebiotic route to nucleotides that can form base pairs that are similar to those formed by the canonical nucleobases (Fig. 1a). Nucleotides spontaneously form when BA is mixed in water with one equivalent of R5P at 20 °C (without the need for drying). The reaction is surprisingly efficient with BA+R5P conjugates exceeding 80% in the unpurified (crude) reaction mixture after 24 h and with significant yields for reactions performed over the range of pH 3–11 (Fig. 1b and Supplementary Fig. 1). Reactions between melamine and R5P are also productive, forming melamine+R5P conjugates at yields ranging from 33 to 55% when the reaction was carried out for 24 h at 65 °C (Fig. 1c). Glycosylation of melamine with R5P was observed from pH 3 to 9 and at 20 °C (Supplementary Figs 2 and 3). Glycosylation of BA and melamine by (unphosphorylated) ribose was also found to occur spontaneously in water, producing four nucleoside isomers for each reaction (Supplementary Figs 4 and 5). The robustness (for example, wide pH and temperature range) and good yields for both nucleoside and nucleotide formation with BA and melamine are noteworthy among model prebiotic reactions, especially considering that none of the four canonical nucleobases form nucleosides in detectable yields when heated with R5P in water (Supplementary Fig. 6). On the contrary, the canonical nucleosides and nucleotides of RNA are thermodynamically disfavoured (but kinetically stable) in water^29^.

**Figure 2:**
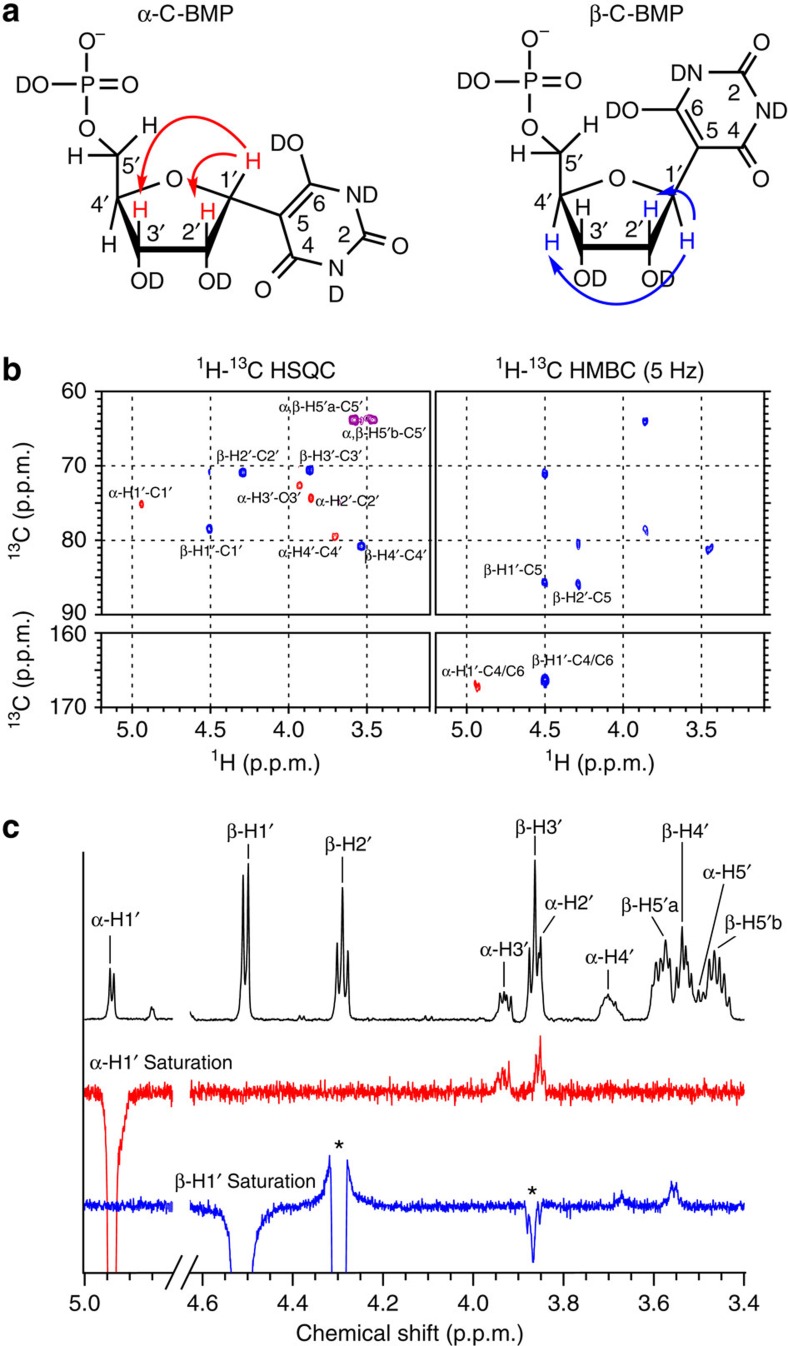
NMR characterization of C-BMP nucleotides. (**a**) Chemical structure of α-C-BMP and β-C-BMP with arrows indicating through-space proton–proton magnetization transfer (ROE) as shown in **c**. (**b**) Heteronuclear single-quantum correlation (HSQC) and heteronuclear multiple bond correlation (HMBC) spectra showing ^1^H-^13^C couplings for α-C-BMP and β-C-BMP. ^1^H-^13^C correlation observed between H1′ and C5 of β-C-BMP and H1′-C4/C6 of α-C-BMP and β-C-BMP in the HMBC as well as the absence of H5-C5 correlations in the HSQC support the C-nucleoside assignment. α-Anomer cross peaks are shown in red, β-anomer cross peaks in blue and overlapping cross peaks in purple. ^1^H Correlation spectroscopy (COSY) spectrum of a mixture of C-BMP nucleotides is provided in the Supplementary Information. (**c**) ^1^H NMR and 1D ROE spectra of a solution containing a 1:2 ratio of α-C-BMP to β-C-BMP. (Top) ^1^H NMR spectrum with resonance assignments as indicated in **a**. (Middle) Irradiation of the H1′ of α-C-BMP results in through space magnetization transfer to the H2′ and H3′ of α-C-BMP. (Bottom) Irradiation of the H1′ of β-C-BMP results in through space magnetization transfer to the H4′ of β-C-BMP. * Indicates TOCSY transfer from β-H1′ to β-H2′ and β-H3′.

To characterize the nucleotides formed from the BA reactions with R5P, the products formed in water after 24 h at 20 °C were isolated by column chromatography for further analysis. Two-dimensional (2D) NMR spectroscopy confirmed that the BA+R5P conjugates are C-nucleotides, with a C–C glycosidic bond between ribose and BA (5-ribofuranosyl-C-barbiturate-5′-monophosphate, C-BMP; Fig. 2b and Supplementary Fig. 7). One-dimensional (1D) rotating frame NOE (ROE) spectroscopy confirmed that the β-anomer is preferentially formed in a 67:33 ratio over the α-anomer (Fig. 2c). Similar to our previous report that reactions between 2,4,6-triaminopyrimidine and ribose yield the C-linked β-ribofuranoside as the major product^10^, BA reactions with R5P again demonstrate that the biologically significant β-anomeric sugar form can be preferentially selected through nucleosidation with an alternative nucleobase. Intermediate products suggest that the BA nucleotide-formation reaction proceeds through a Knoevenagel condensation (Supplementary Fig. 8a). We note that β-C-BMP shares a close structural relationship with the C-nucleotide pseudouridine, the most common posttranscriptional modification of RNA in biology^30^.

**Figure 3:**
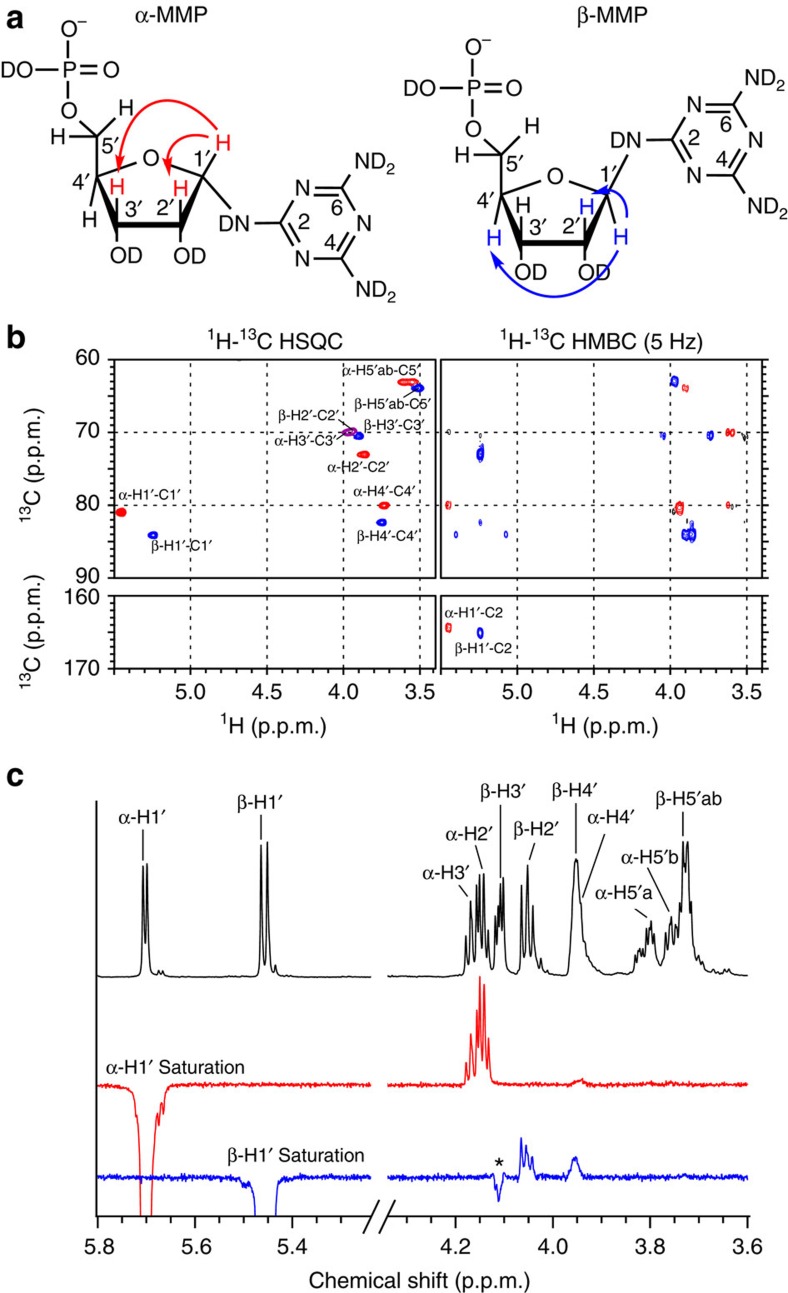
NMR characterization of MMP nucleotides. (**a**) Chemical structure of α-MMP and β-MMP with arrows indicating through-space proton–proton magnetization transfer as shown in **c**. (**b**) HSQC and HMBC spectra showing ^1^H-^13^C couplings for α-MMP and β-MMP. ^1^H-^13^C correlation observed in the HMBC (H1′–C5) of α-MMP and β-MMP show coupling between ribose and melamine. α-Anomer cross peaks are shown in red, β-anomer cross peaks in blue and overlapping cross peaks in purple. ^1^H COSY spectrum of a mixture of MMP nucleotides is provided in the Supplementary Information. (**c**) ^1^H NMR and 1D ROE spectra of a solution containing an approximately equal concentration of α-MMP to β-MMP. (Top) ^1^H NMR spectrum with resonance assignments as indicated in **a**. (Middle) Irradiation of the H1′ of α-MMP results in through space magnetization transfer to the H3′ of α-MMP. (Bottom) Irradiation of the H1′ of β-MMP results in through space magnetization transfer to the H2′ and H4′ of β-MMP. * Indicates TOCSY transfer from β-H1′ to β-H3′.

We next isolated and characterized the melamine+R5P conjugates formed from a reaction between melamine and R5P that was performed at 65 °C over 24 h. 2D NMR analysis of the melamine+R5P conjugates confirmed that glycosylation of melamine occurs at an exocyclic amine (Fig. 3b and Supplementary Fig. 9). Glycosylation proceeds through a reversible Schiff base intermediate (Supplementary Fig. 8b), which is partially stabilized by ring closure, to form two *N*-nucleotides (N-ribofuranosyl-melamine-5′-monophosphate (MMP)). 1D ROE analysis confirms that the α*-* and β-anomers of MMP equilibrate to approximately equal amounts in aqueous solution (Fig. 3c). The stability of the glycosidic bond of exocyclic *N*-linked triaminotriazine nucleosides in water had previously been shown to be quite poor, with total hydrolysis occurring on the order of minutes^27^. The hydrolytic stability of a 5 mM solution of MMP was evaluated to determine the rate of hydrolysis at pH 5 and 5 °C. Under these conditions, the hydrolysis rate was determined to be 3 × 10^−6^ min^−1^, with a half-life of 6 months (Supplementary Fig. 10). Remarkably, the dissociation constant between melamine and R5P was found to be 3.7 mM. For comparison, the work of Miller and co-workers^20^ revealed that the *K*_d_ for the ribonucleoside of urazole is 700 mM at 5 °C, whereas the *K*_d_ for the ribonucleoside of uracil is estimated to be around 700 M. Thus, the equilibrium of nucleoside formation between melamine and ribose could be several orders of magnitude more favourable than that of the extant nucleobases.

### MMP and C-BMP form supramolecular assemblies

**Figure 4:**
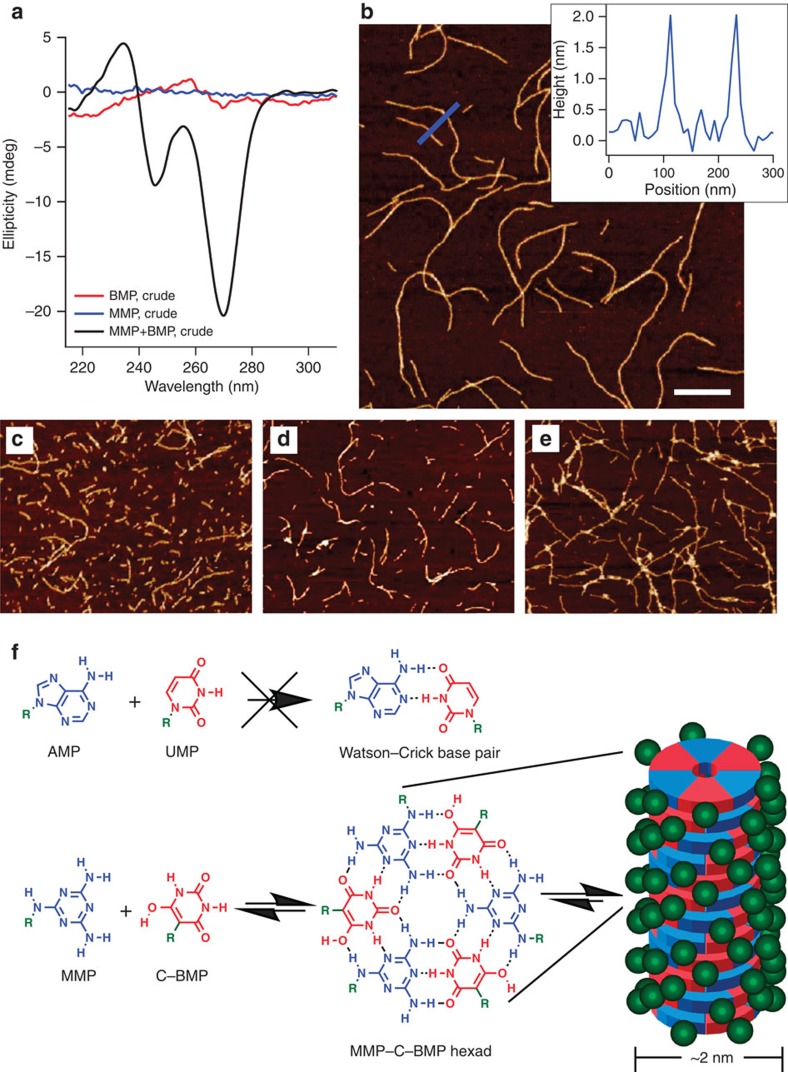
Nucleotides assemble into supramolecular polymers. (**a**) CD spectra of melamine-R5P and BA-R5P crude reaction mixtures, separate and combined at 5 °C. Black curve is mixture of melamine-R5P and BA-R5P crude reaction mixtures; red curve is BA-R5P crude reaction mixture; blue curve is melamine-R5P crude reaction mixture. Ultraviolet spectra associated with CD spectra are provided in Supplementary Fig. 11. (**b**–**e**) AFM topographic images of (**b**) mixture of melamine-R5P and BA-R5P crude reaction mixtures, (**c**) mixture of purified C-BMP and MMP, (**d**) purified C-BMP mixed with melamine, (**e**) purified MMP mixed with BA. Inset in **b** shows height measurements of blue line in image. Scale bar in **b** is 300 nm, and all AFM images are shown at the same magnification. (**f**) Chemical structures of MMP and C-BMP and their association into 2 nm wide stacked hexads. The green R and green spheres indicate R5P. The similarity of AMP to MMP and UMP to C-BMP, and the inability of these canonical nucleosides to assemble in aqueous solution is also illustrated. All solutions contained 1 M NaCl.

We next tested the ability of MMP and C-BMP to exhibit base pairing in aqueous solution. Base pairing is *not* exhibited by the canonical mononucleotides^31^, but such a property would have been advantageous for the prebiotic mutual selection and co-localization of base-pairing nucleotides, particularly if proto-biopolymers emerged from a complex ‘prebiotic soup'^32^,^33^. The crude products of melamine and BA reactions with R5P were mixed and the mixtures were analysed by circular dichroism (CD) spectroscopy as an initial test of whether melamine and BA nucleotides can form chiral assemblies even without purification. To maximize assembly of the two heterocycles, solution pH was adjusted to between pH 4 and 5. This pH range was chosen due to p*K*_a_ considerations of the parent heterocycles (BA p*K*_a_=4; melamine p*K*_a_=5), which would be expected to minimize the difference in relative ionization between the bases^34^. This mixture of nucleotides, side products and unreacted starting materials from the two reactions (50 mM each in total BA and melamine) exhibits a substantial CD signal, whereas separately the crude products of each reaction exhibit either no CD signal (the melamine-R5P reaction) or a much lower CD signal and with a different wavelength profile (the BA-R5P reaction; Fig. 4a). The loss of the CD signal upon heating the mixture of the crude reactions above 30 °C, and return of signal upon cooling to 5 °C (Supplementary Fig. 11a) indicates the reversible formation of non-covalent assemblies.

The assemblies formed upon combining the crude reaction mixtures were imaged by atomic force microscopy (AFM), which revealed linear supramolecular polymers with diameters of ca 2 nm (Fig. 4b). These structures are fully consistent with the presence of stacked hydrogen-bonded hexads with paired BA and melamine bases (Fig. 4f)^23^, assemblies that have been observed previously with analogous molecules that also form hexads^10^,^35^. The length of these supramolecular polymers (typically >1 μm) indicates that tens of thousands of heterocycles are paired within a single assembly. When solutions containing purified MMP and C-BMP were combined and analysed by AFM, supramolecular polymers with a diameter of 2 nm were also observed (Fig. 4c and Supplementary Fig. 11b). As can be seen in Fig. 4b,c, the assemblies formed from purified MMP and BMP are noticeably shorter than those formed upon combining the crude reaction mixtures. This observation is consistent with length being limited by greater peripheral charge^36^, an effect that is expected to be greater for supramolecular polymers formed from the purified nucleotides than it is for assemblies containing both nucleotides and the parent heterocycles (that is, those present in the crude reaction mixtures).

C-BMP and MMP nucleotides also form water-soluble supramolecular assemblies when mixed with free melamine and free BA, respectively. These C-BMP-melamine and MMP-BA assemblies were visualized by AFM (Fig. 4d,e and Supplementary Fig. 11c,d), revealing 2 nm fibres that are again indicative of stacked hexad assemblies. Although free BA and free melamine form insoluble precipitates when mixed in aqueous solution (Supplementary Fig. 12), the steric bulk and charge provided by conjugation with R5P on one nucleobase favours the formation of the water soluble, linear assemblies of stacked hexads^35^.

### Supramolecular assemblies preferentially incorporate β-MMP

**Figure 5:**
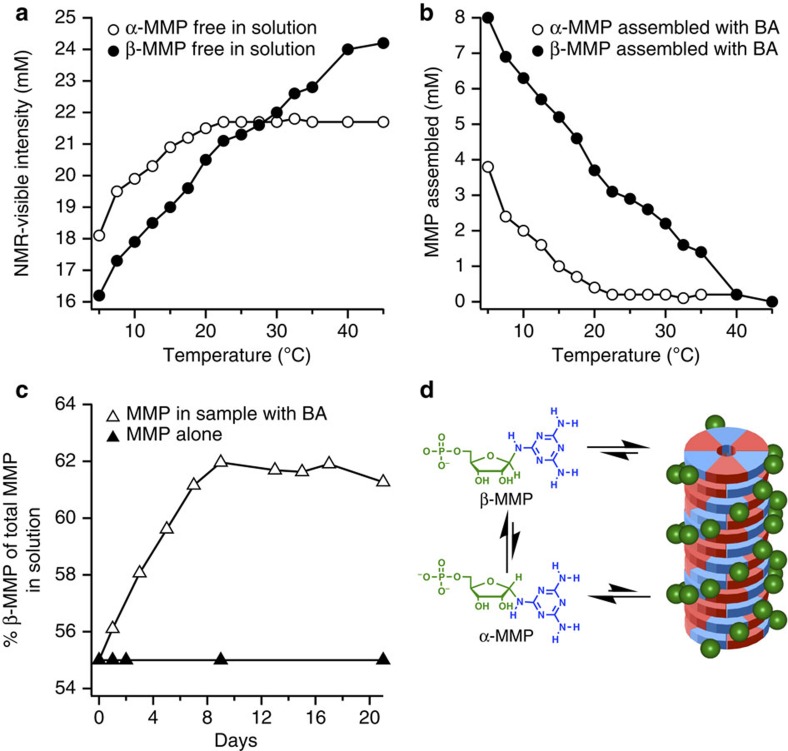
MMP assembly and anomerization in the presence of BA. (**a**) Plot of NMR visible resonance intensity of unassembled α-MMP and β-MMP as a function of temperature in a solution containing 50 mM MMP and BA. (**b**) Plot showing fraction of both MMP anomers assembled at various temperatures (plot generated by subtracting data shown in **a** from measured total concentration of α-MMP and β-MMP in each sample). (**c**) Plot showing the change in anomeric ratio (by percent) of β-MMP as a function of time in solutions containing both MMP and BA, or MMP alone. Samples were maintained at 5 °C during the experiment and diluted just prior to analysis to disassemble MMP in order to enable quantification of the total MMP in solution by NMR. (**d**) Schematic showing preferential assembly (stacked hexads) and anomerization. All solutions contained 0.3 M NaCl.

^1^H-NMR spectroscopy was used to further characterize C-BMP-melamine and MMP-BA assemblies (Supplementary Figs 13 and 14). Nucleotides incorporated into these supramolecular assemblies exhibit extreme ^1^H NMR line broadening (to baseline), which render them invisible to solution state NMR spectroscopy. In contrast, free nucleotides that exist in equilibrium with the assemblies exhibit virtually no change in ^1^H resonance chemical shift or line width. Quantitative analysis of ^1^H resonance intensity can be used to determine the fraction of free nucleotides, and subtraction of these values from the known total concentration of nucleotides in a sample reveals the concentration of assembled nucleotides^35^. Analysis of ^1^H spectra of solutions containing MMP+BA, 50 mM in nucleotide and heterocycle, showed temperature-dependent assembly of nucleotides from 5 to 40 °C (Fig. 5a,b and Supplementary Fig. 14). Unexpectedly, the preferential incorporation for β-MMP over α-MMP was observed at all temperatures where assemblies are present. In particular, a twofold preference for β-MMP incorporation is observed at 5 °C, and only incorporation of β-MMP at 20 °C (Fig. 5b).

As the β-anomer is selectively incorporated into the assemblies over the α-anomer, and anomerization of MMP occurs in the solution (see the Methods for details), we next explored if supramolecular assembly will affect the anomeric ratio of MMP. As noted above, at equilibrium, a solution of MMP contains a mixture of α- and β-anomers (45% α and 55% β), however, when 50 mM MMP was incubated with 50 mM BA at 5 °C, we observe the conversion of α-MMP to β-MMP, a conversion that reached a maximum of 63% β-MMP after 8 days (Fig. 5c). This conversion was not observed when BA was omitted or when MMP was below the minimal concentration required for MMP-BA assembly. Therefore, we have found that the assemblies formed with BA preferentially select/stabilize the β-anomer of MMP, and as a result, with anomerization, the assemblies enrich β-MMP (Fig. 5d). These observations indicate that solutions containing MMP and BA assemblies will change overtime in both molecular (that is, enrichment of β-MMP) and supramolecular composition (that is, more MMP and BA assembled).

## Discussion

The data presented here demonstrate the efficient single-step syntheses of complementary nucleosides and nucleotides, starting with the plausible proto-nucleobases melamine and BA and ribose or R5P. Although R5P forms an exocyclic N-glycosidic bond with melamine (MMP) and R5P forms a C-nucleotide with BA (C-BMP), both of these nucleotides favour their β anomers. Specifically, for the free nucleotides in solution, the β and α anomers of C-BMP exist at equilibrium in a 67:33 ratio, and for MMP the β-anomer is favoured in a 55:44 ratio over the α-anomer. Perhaps coincidentally, extant life uses the β-anomeric form of ribonucleotides, and our observations indicate that this form may have been enriched on the early Earth for β-ribonucleotides for which glycosidic bond anomerization is under equilibrium control. This nucleotide structural preference is apparently not limited to the nucleotides of melamine and BA, as previous studies have also revealed that nucleosides formed by drying and heating ribose with two other pyrimidines, 2-pyrimidinone^21^,^37^ and 2,4,6-triaminopyrimidine^23^, also favour the β-anomer.

Unlike the mononucleotides of extant RNA, we observe that MMP and C-BMP will pair as monomers in aqueous solution, with each other and with their unmodified pairing partners (that is, MMP with C-BMP, MMP with BA, and C-BMP with melamine), producing, in all cases, supramolecular structures that indicate the highly efficient stacking of H-bonded hexads that are themselves composed of Watson–Crick-like base pairs. This pairing and stacking is sufficiently robust to drive assembly in the presence of the side products and unreacted starting materials of the crude nucleotide reactions. Based on these observations, and because C-BMP and MMP structurally resemble two nucleotides found in life today (UMP and AMP, respectively) and have been reported to pair with extant, complementary nucleobases^38^,^39^, it is tempting to speculate that these heterocycles could represent ancestral nucleotides of the contemporary genetic polymers. In particular, the ability for C-BMP and MMP to form *noncovalent* supramolecular assemblies could have facilitated the prebiotic localization, organization and subsequent linking of these (or similar) nucleotides into *covalent* polymers that were then capable of storing and transferring information (for example, by templating the formation of sequence-specific assemblies for the polymerization of additional monomers^40^).

The ability of C-BMP and MMP to form supramolecular assemblies might have also facilitated the emergence of early RNA-like polymers by selecting nucleotides with sugars (or earlier trifunctional linkers^19^) that were structurally compatible with the assemblies and their subsequent coupling into covalent polymers. In the present study, we have, for practical reasons, used D-ribose and D-R5P for our nucleoside and nucleotide reactions with melamine and BA, but L-ribose or L-R5P would exhibit equivalent reactivity with these two heterocycles. Nevertheless, it has been often postulated that a racemic mixture of nucleotides would have inhibited the prebiotic synthesis of RNA polymers^41^, and so the question of how the present system might address this challenge deserves some discussion. Although we have not shown chiral nucleotide selection, in the current study we have demonstrated that the β-anomer of MMP is enriched in supramolecular assemblies over the α-anomer of MMP, and this selection leads to a detectable increase in the ratio of the β-anomer over the α-anomer of MMP in the entire solution (presumably due to anomerization and selective stabilization by the assembly). As a recent example of the ability of supramolecular polymers to promote local chiral resolution, Aida and co-workers demonstrated that racemic solutions of chiral macrocycles self-sort into homochiral supramolecular polymers^42^. It is therefore possible that supramolecular assemblies, formed by nucleotides with different sugars, including different anomers and enantiomers, could have been selectively enriched in individual supramolecular assemblies before polymerization. Current investigations of this possibility are actively being pursued in our laboratory.

## Methods

### Materials

Melamine and BA were purchased from Acros Organic, D-R5P disodium salt and D-ribose were purchased from Sigma-Aldrich. All chemicals were used as received.

### Synthesis of C-BMP

BA (2.5 mmol) and R5P (2.5 mmol) were dissolved in 5 ml of H_2_O and the pH was adjusted to 9 with NaOH (unless otherwise noted). The solution was stirred for 24 h at 20 °C, at which time a clear, pale yellow solution was present. This solution is referred to as the crude BA-R5P reaction mixture. To isolate C-BMP, the crude reaction mixture was loaded onto a gravity column containing QAE Sephadex A-25 anion exchange media and eluted with a gradient of NH_4_HCO_3_ buffer (pH 9.4) from 50 mM to 0.5 M. The fractions containing product were lyophilized, redissolved in water and pooled. When the reaction was performed at pH 9, yield of C-BMP was 82% (α-C-BMP 22%, β-C-BMP 60%); ^1^H NMR of the crude reaction mixture is shown in Fig. 1b. HRMS (*m*/*z*): [M]^−^ calculated for C_9_H_12_N_2_O_10_P^−^, 339.0235; found, 339.0243. Ultraviolet–visible: (50 mM NaH_2_PO_4_, pH 7) *λ*_max_=260 nm; *ɛ*_260_=23,000 mol l^−1^ cm^-1^.

*α-C-BMP*. ^1^H NMR (500 MHz, D_2_O): *δ* 4.94 (d, *J*=4.2 Hz, H1′), 3.85 (dd, *J*=4.2, X Hz, H2′), 3.93 (dd, *J*=4.1, 3.5 Hz, H3′), 3.70 (m, H4′), 3.59 (m, H5′a), 3.46 (m, H5′b); ^13^C NMR (126 MHz, D_2_O): *δ* 167.1 (C4/C6), 152.7 (C2), 85.5 (C5), 79.5 (d, *J*=8.2 Hz, C4′), 75.1 (C1′), 74.3 (C2'), 72.7 (C3′), 63.6 (d, *J*=4.4 Hz, C5′).

*β-C-BMP*. ^1^H NMR (500 MHz, D_2_O): *δ* 4.51 (d, *J*=5.7 Hz, H1′), 4.29 (t, *J*=5.7 Hz, H2′), 3.86 (t, *J*=6.2 Hz, H3′), 3.54 (m, H4′), 3.58 (m, H5′a); 3.46 (m, H5′b); ^13^C NMR (126 MHz, D_2_O): *δ* 166.4 (C4/C6), 153.1 (C2), 85.7 (C5), 80.8 (d, *J*=7.9 Hz, C4′), 78.6 (C1′), 70.9 (C2′), 70.5 (C3′), 63.8 (d, 4.6 Hz C5′).

### Synthesis of MMP

Melamine (1 mmol) and R5P (1 mmol) were dissolved in 5 ml of H_2_O and the pH was adjusted with HCl to pH 5, unless otherwise noted. The solution was stirred at 65 °C for 24 h, at which point a clear, light brown solution was present. This solution is referred to as the melamine-R5P crude reaction mixture. To isolate MMP, the crude reaction mixture was loaded onto a gravity column containing SP Sephadex C-50 cation-exchange media and eluted with NH_4_OAc buffer, 50 mM, pH 4.3. The fractions containing product were lyophilized, redissolved in water and pooled. When the reaction was performed at pH 5, yield of MMP was 55% (α-MMP 26.4%, β-MMP 28.6%); ^1^H NMR of the crude reaction mixture is shown in Fig. 1c. HRMS (*m*/z): [M]^−^ calculated for C_8_H_14_N_6_O_7_P^−^, 337.0667; found, 337.0659. Ultraviolet–visible: (50 mM NaH_2_PO_4_, pH 7) *λ*_max_=235 nm; *ɛ*_235_=5,500 mol l^−1^ cm^−1^.

*α-MMP*. ^1^H NMR (500 MHz, D_2_O): *δ* 5.46 (d, *J*=4.1 Hz, H1′), 3.97 (dd, *J*=4.7, 1.9 Hz, H3′), 3.94(dd, *J*=4.7, 4.1 H2′), 3.74 (m, H4′), 3.64 (m, H5′a), 3.55 (m, H5′b); ^13^C NMR (126 MHz, D_2_O): *δ* 164.4 (C2), 164.2 (C4/C6), 81.0 (C1′), 79.5 (d, *J*=8.2 Hz, C4′), 70.0 (C3′), 69.9 (C2'), 63.4 (d, *J*=5.3, C5′).

*β-MMP*. ^1^H NMR (500 MHz, D_2_O): *δ* 5.25 (d, *J*=6.6 Hz, H1′), 3.89 (dd, *J*=5.4, 3.2 Hz, H3′), 3.85 (dd, *J*=6.6, 5.4 Hz, H2′), 3.75 (m, H4′), 3.49 (m, H5′a,b); ^13^C NMR (126 MHz, D_2_O): *δ* 165.1 (C2), 164.9 (C4/C6), 84.2 (C1′), 82.3 (d, *J*=8.0 Hz, C4′), 73.1 (C2′), 70.5 (C3′), 64.2 (d, 4.5 Hz, C5′).

### General assembly protocol

All solutions were 50 mM in each heterocycle. Final concentration of solutions containing crude reaction mixtures of either nucleotide was 50 mM in total heterocycle (that is, both unmodified and glycosylated). Upon combining melamine and BA nucleotides and/or parent heterocycles, the pH of solution was adjusted with HCl or NaOH to pH 4.5–5. This pH range was chosen as it would be expected to maximize assembly of the heterocycles due to p*K*_a_ considerations (p*K*_a_ of protonated melamine is 5 and the p*K*_a_ of BA is 4)^34^. All solutions contained either 0.3 or 1.0 M NaCl as noted. Anomerization experiments were performed with MMP that was first incubated at 5 °C for 24 h to enable the anomeric ratio to equilibrate under the conditions tested. Variable temperature NMR experiments were performed on samples containing MMP and BA that were mixed and stored at 5 °C for 15 h before analysis to ensure that the association between MMP and BA had reached equilibrium. Stock solutions of nucleotides and crude reaction mixtures were stored at −20 °C.

### Spectroscopic analysis of the supramolecular assemblies

CD analysis was carried out on a Jasco J-810 CD spectrometer equipped with a six-cell Quantum Northwest peltier temperature controller. Strain-free 0.01 mm demountable cells from Starna were used for all CD analysis. ^1^H NMR spectra were collected on a Bruker DRX-500 NMR and were the sum of 32 transients. All NMR samples were D_2_O exchanged and lyophilized before analysis in D_2_O with an internal standard of 3-(trimethylsilyl)-2,2',3,3'-tetradeuteropropionic acid at 1.11 mM. Variable temperature NMR experiments were performed by gradually heating the solutions in order to disassemble supramolecular assemblies present, or slow cooling to enable reassembly, followed by short incubation at the desired temperature to allow for the assemblies to reach equilibrium. To analyse anomerization of MMP over time, a sample of MMP and BA (50 mM each) was stored at 5 °C, and at specified time points, samples were removed and diluted with D_2_O to 5 mM in MMP total (a concentration below the minimal assembly concentration) and analysed by NMR at 20 °C

### Atomic force microscopy

Imaging was performed with a Nanoscope IIIa (Digital Instruments) in tapping mode, using Si tips (MicroMash, 16 N m^−1^). A freshly cleaved mica substrate was pre-activated by incubation with a solution of 20 mM MgCl_2_ that was then rinsed with water and dried under N_2_ (*g*). A 3-μl sample (initially stored on ice) was deposited on the mica substrate and spread with N_2_ (*g*) followed by the addition of 50 μl of cold water to remove sample that was not adsorbed to the mica surface and quickly dried again with N_2_ (*g*).

### Analytical HPLC

Analysis of nucleotide samples was performed by running a linear gradient of 90% acetonitrile/10% water to 50% acetonitrile/H_2_O for 7 min, followed by isocratic 90% aqueous acetonitrile for 6 min on a Waters XBridge Amide (150 × 2.5 × 3.5 μm^3^) column. Analytical HPLC analysis of nucleoside samples was performed by running a linear gradient of 85% acetonitrile/15% water to 40% acetonitrile/60% water over 25 min on a Waters XBridge Amide (150 × 2.5 × 3.5 μm^3^) column.

## Additional information

**How to cite this article:** Cafferty, B. J. *et al*. Spontaneous formation and base pairing of plausible prebiotic nucleotides in water. *Nat. Commun.* 7:11328 doi: 10.1038/ncomms11328 (2016).

## Supplementary Material

Supplementary Information Supplementary Figures 1-14
