# Supplementary material for: Spontaneous formation and base pairing of plausible prebiotic nucleotides in water
Source: Nat Commun. 2016 Apr 25;7:11328. doi: 10.1038/ncomms11328 (PMC4848480; doi:10.1038/ncomms11328)
Supplement: Supplementary Information — Supplementary Figures 1-14 [file ncomms11328-s1.pdf]

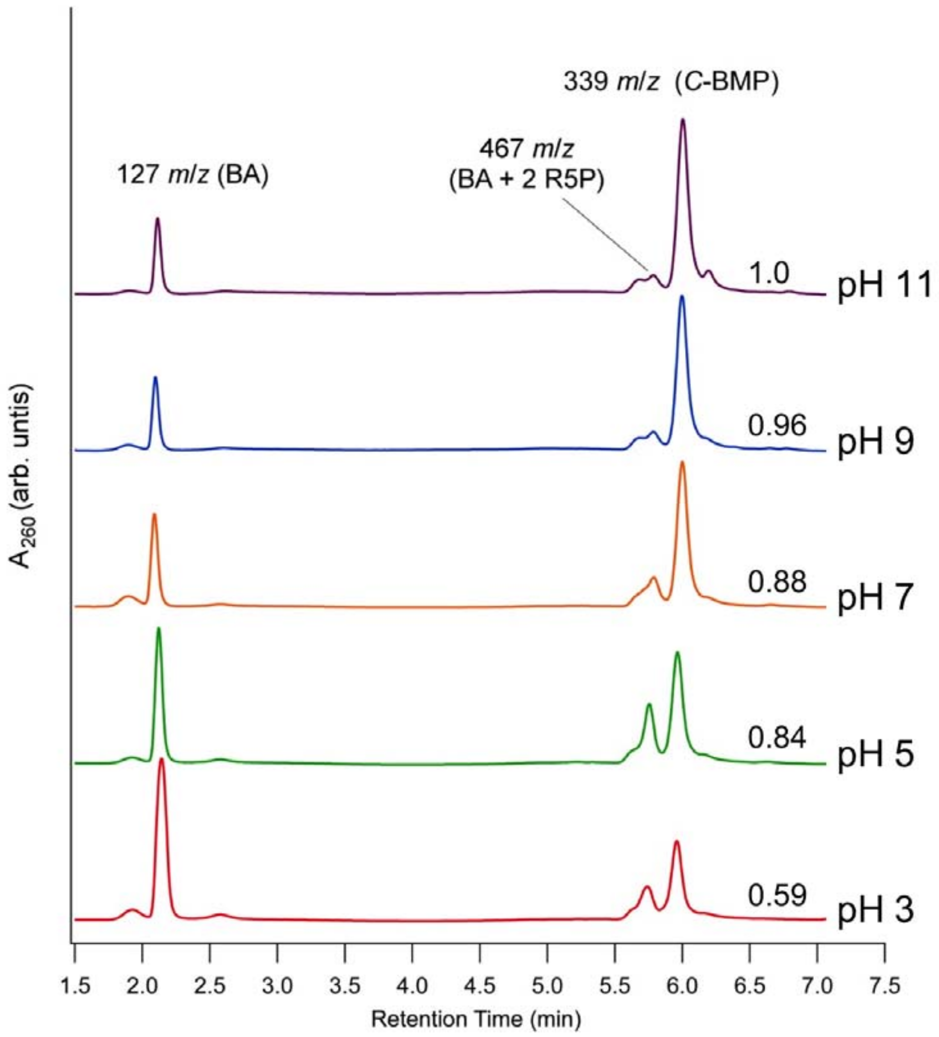

Supplementary Figure 1| HPLC chromatograms of samples from BA+R5P reactions performed at various pH. Chromatograms of samples of BA and R5P after reacting a solution originally at 500 mM in each molecule at 20°C in water at various pH for 24 h. Peaks are labeled with *m*/*z* values (negative ion mode) obtained by simultaneous MS and UV monitoring of LC. The *m*/*z* values listed correspond to BA, 127; BA with one closed-ring ribose-5-phosphate conjugate, 339; and BA with two linear ribose-5-phosphate conjugates, 467. Numbers near *C*-BMP peaks represent relative yields of *C*-BMP between experiments, based on integration of *C*-BMP peak areas and normalization to highest yield of this set of experiments. The pH was adjusted with NaOH or HCl. Reactions were performed in triplicate.

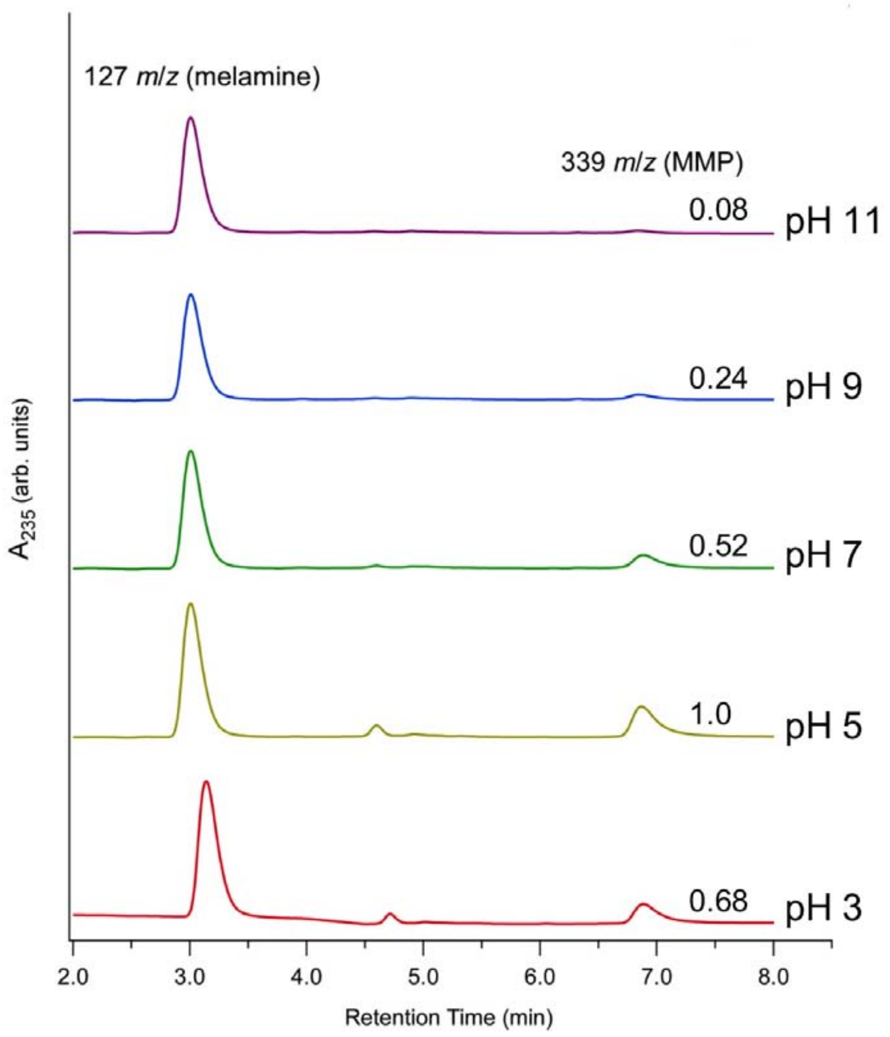

Supplementary Figure 2| HPLC chromatograms of samples from melamine+R5P reactions performed at various pH. Chromatograms of samples of melamine (1 mmol) and R5P (1 mmol) after reacting at 65°C in 5 ml of water at various pH for 24 h. Peaks are labeled with *m*/*z* values (positive ion mode) obtained by simultaneous MS and UV monitoring of LC. The *m*/*z* values listed correspond to melamine, 127; and melamine with one closed-ring ribose-5-phosphate conjugate, 339. Numbers near MMP peaks represent relative yields of MMP between experiments, based on integration of MMP peak areas and normalization to highest yield of this set of experiments. The pH was adjusted with NaOH or HCl. Melamine was only partially soluble at pH 7 and above, to obtain representative samples well-mixed slurries were solubilized by dilution prior to analysis. Reactions were performed in triplicate.

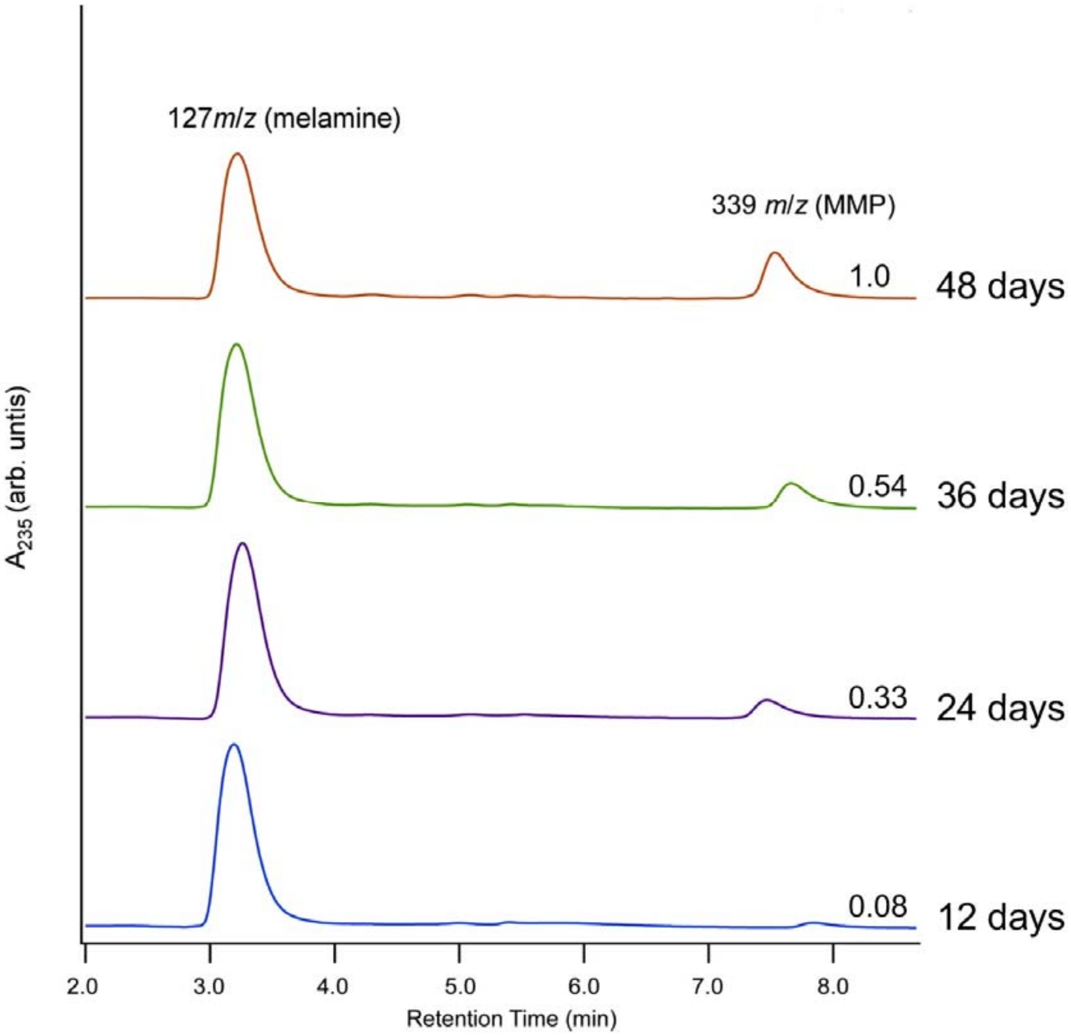

Supplementary Figure 3| HPLC chromatograms of samples from a melamine+R5P reaction performed at 20°C over 48 days. Samples were taken at various time points over 48 days from a reaction of melamine (0.5 mmol) and R5P (0.5 mmol) in 5 ml of water at 20°C and pH 7 (same reaction conditions performed for *C*-BMP synthesis). The pH of each reaction mixture was adjusted with HCl. Numbers near MMP peaks represent relative yields of MMP between experiments, based on integration of MMP peak areas and normalization to the highest yield of this set of experiments. Melamine was not fully soluble under these conditions (20°C, pH 7), to obtain representative samples well mixed slurries were solubilized by dilution prior to analysis. Peaks are labeled with *m/z* values (positive ion mode) obtained by simultaneous MS and UV monitoring of LC.

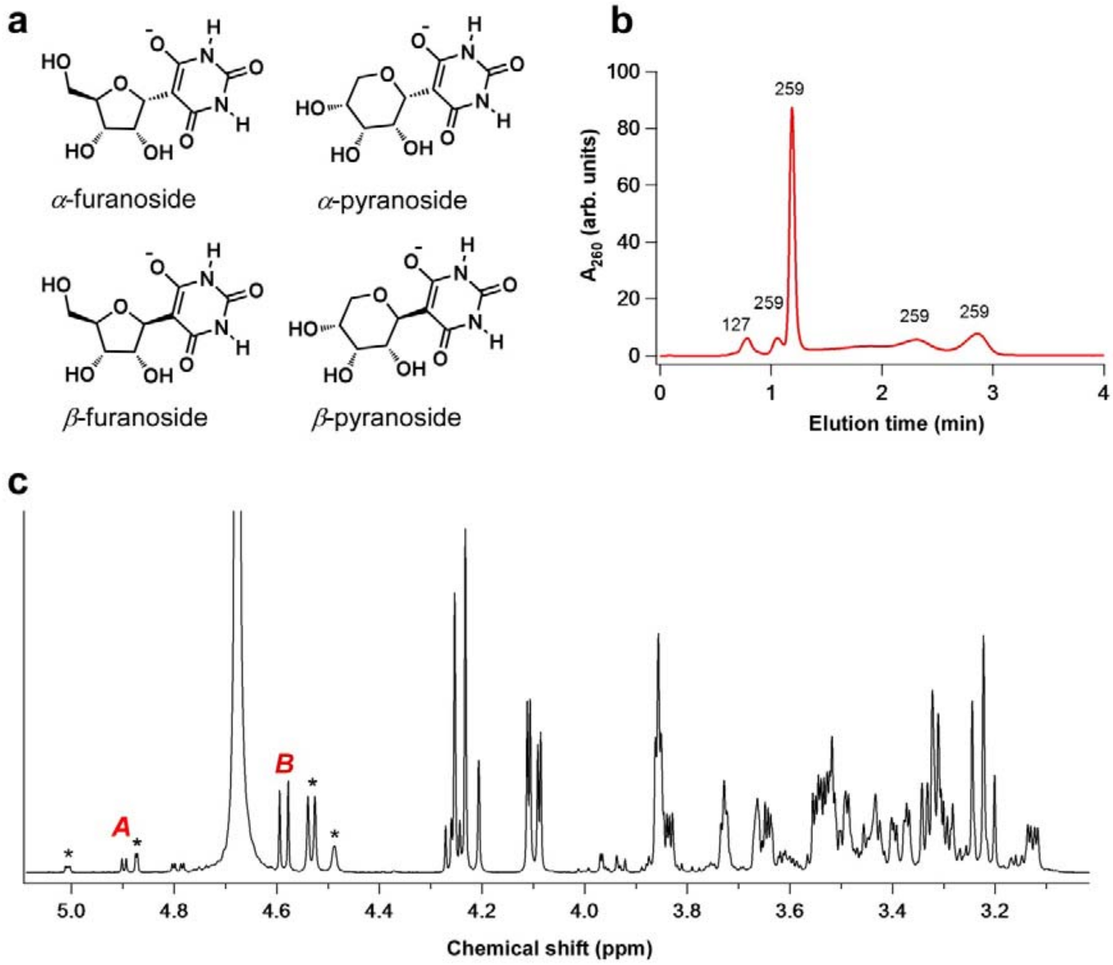

Supplementary Figure 4| Glycosylation of BA by ribose in water. a, Chemical structures of BA+ribose conjugates that are formed by reacting BA with ribose. **b**, HPLC chromatogram of samples of BA and ribose after reacting at 20°C for 24 h in water. Peaks are labeled with *m/z* values obtained by simultaneous MS (negative ion mode) and UV absorption monitoring of LC. The *m/z* values listed correspond to the BA heterocycle, 127; and four peaks with the mass of BA with a closed-ring ribose conjugate, 259. Integration of these peaks was used to determine the total yield of nucleoside product to be 90%. **c**, ^{1}H NMR of the BA+ribose reaction depicting the nonexchangeable ribose protons. The anomeric protons of the four ribose sugar forms are labeled (*). From comparative analysis with the BMP anomers the *α* and *β* anomeric protons of BA-ribofuranosides were assigned and are labeled *A* and *B*, respectively. The two doublets at 4.8 ppm are tentatively assigned to a doubly ribosylated BA, an assignment that is supported by the observation of a product with the expected mass in BA-ribose reactions that increases in samples with higher proportions of ribose to BA. Reactions initially contained 500 mM ribose and 500 mM BA, and were adjusted to pH 9 with NaOH. MS analysis was performed in negative ion mode.

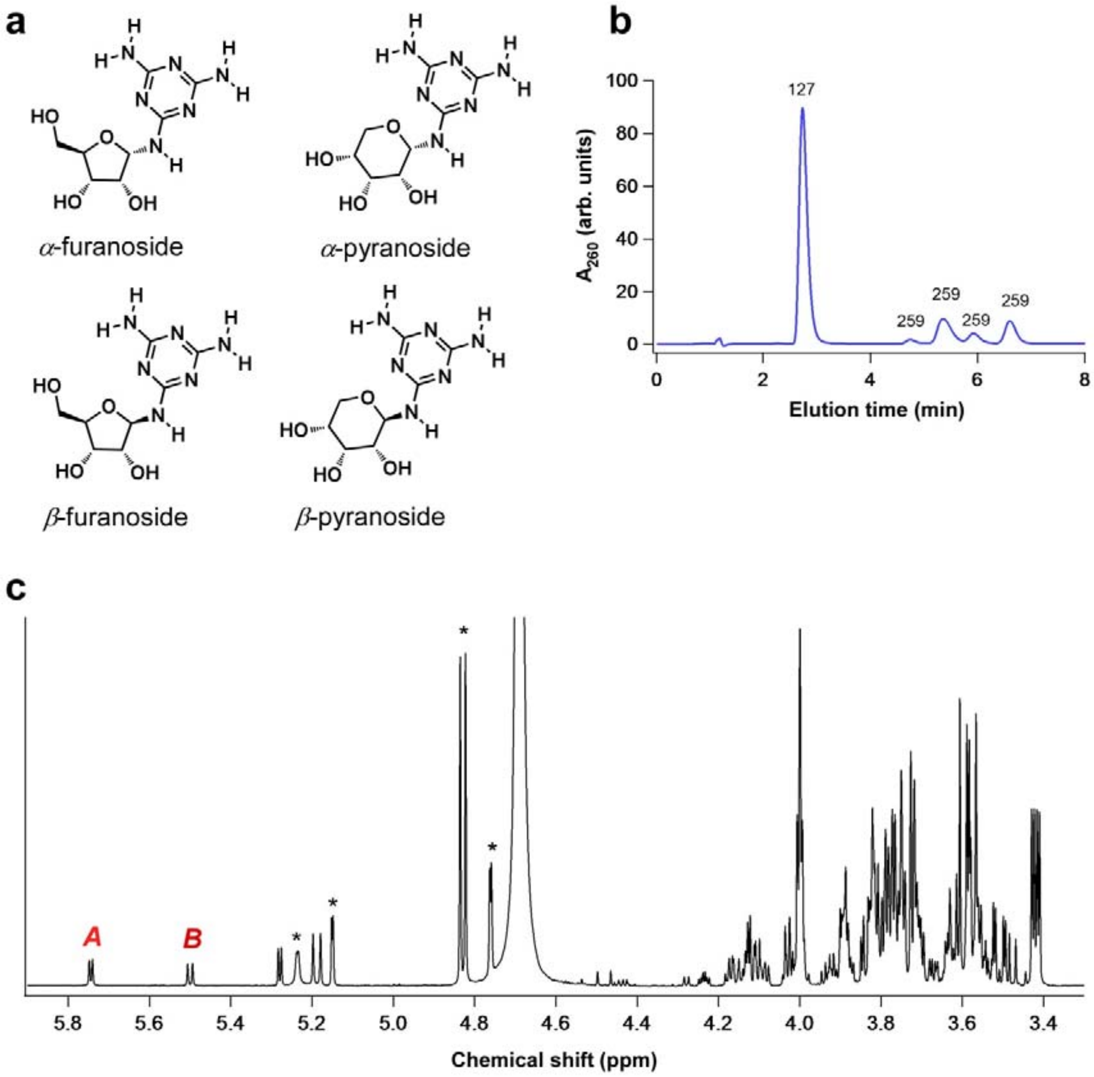

Supplementary Figure 5| Glycosylation of melamine by ribose in water. a, Chemical structures of melamine+ribose conjugates that can be formed by reacting melamine with ribose. **b**, HPLC chromatogram of samples of melamine and ribose after reacting at 65°C for 24 h in water. Peaks are labeled with *m/z* values obtained by simultaneous MS (positive ion mode) and UV absorption monitoring of LC. The *m/z* values listed correspond to the melamine heterocycle, 127; and four peaks with the mass of melamine with a closed-ring ribose conjugate, 259. Integration of these peaks was used to determine the total yield of nucleoside product to be 28%. **c**, ^{1}H NMR of the melamine+ribose reaction depicting the nonexchangeable ribose protons. The anomeric protons of the four ribose sugar forms are labeled (*). From comparative analysis with the anomers of MMP, the anomeric protons of the α and β melamine-ribofuranosides and, by exclusion, the α and β melamine-ribopyranosides are labeled *A*, *B*, *C*, and *D* respectively. Reactions initially contained 200 mM ribose and 200 mM melamine, and were adjusted to pH 5 with HCl. MS analysis was performed in positive ion mode.

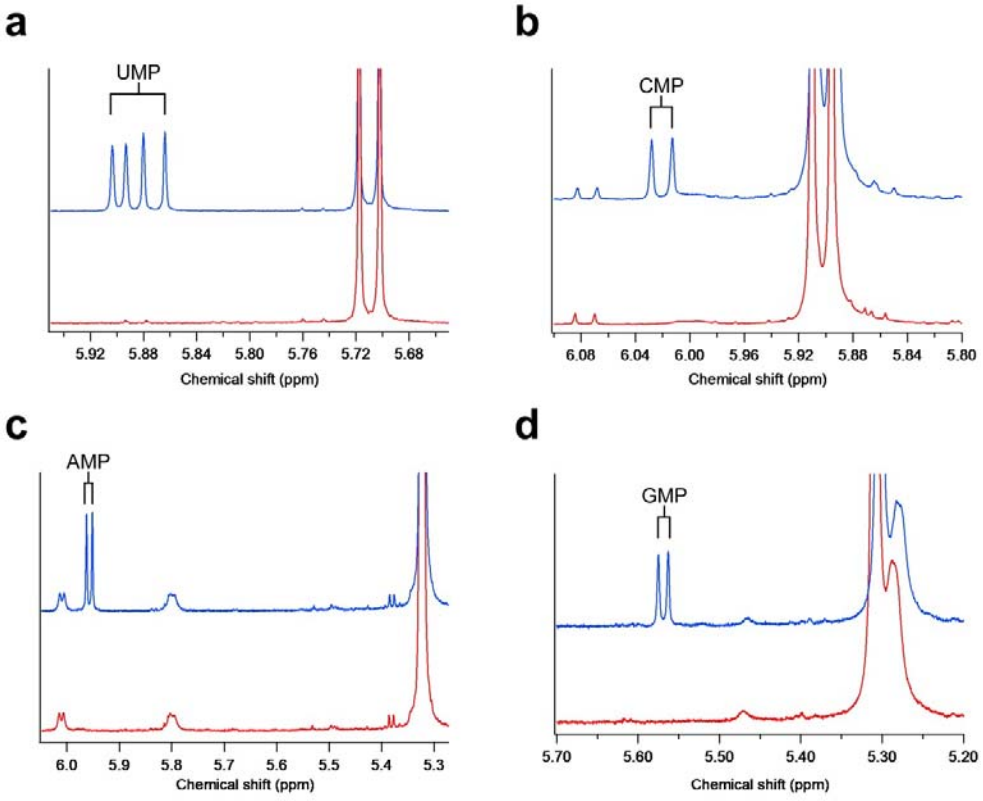

Supplementary Figure 6| Canonical RNA nucleobases do not react with R5P to produce nucleotides in water. Solutions containing 200 mM R5P and 200 mM nucleobase (uracil, cytosine, adenine or guanine) were heated at 65°C in water at pH 7. After 24 h the solutions were lyophilized, resuspended in D_2O, and analyzed by ^1H NMR with (blue traces) or without (red traces) addition of the corresponding nucleotide at 5 mM as an internal standard. Select regions of these NMR spectra are shown for reactions with **a**, uracil; **b**, cytosine; **c**, adenine; **d**, and guanine. The appearance of peaks (labeled) with the addition of the nucleotide internal standard — where no peaks were visible without the addition — reveals that the canonical nucleotides are not produced under these conditions. We note that cytosine, adenine and guanine were not fully soluble under the reaction conditions performed here. Reactions were performed in triplicate.

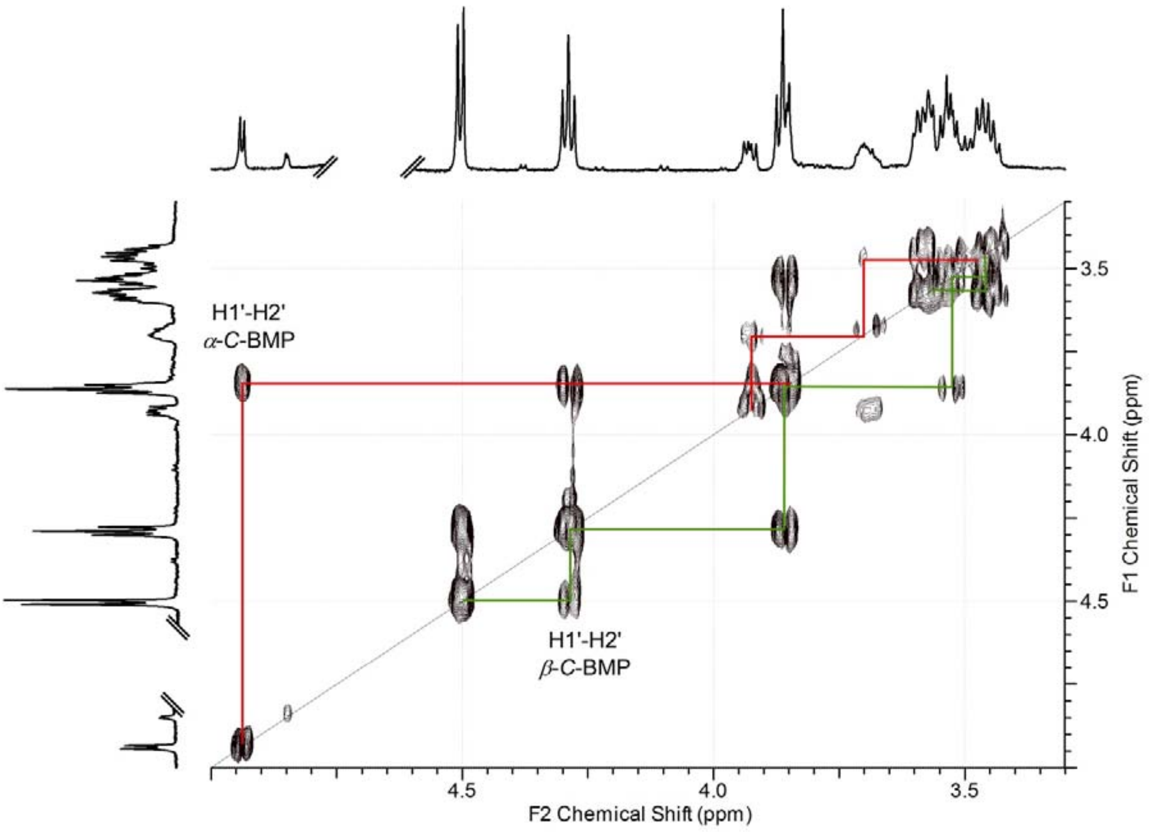

Supplementary Figure 7| ^{1}H and 2D COSY spectra of *C*-BMP. Along with HSQC and HMBC spectra present in Figure 2 of main text, COSY was used to confirm proton assignments of *α*-*C*-BMP and *β*-*C*-BMP. Red lines connect the cross-peaks of *α*-*C*-BMP and green lines connect the cross-peaks of *β*-*C*-BMP.

a

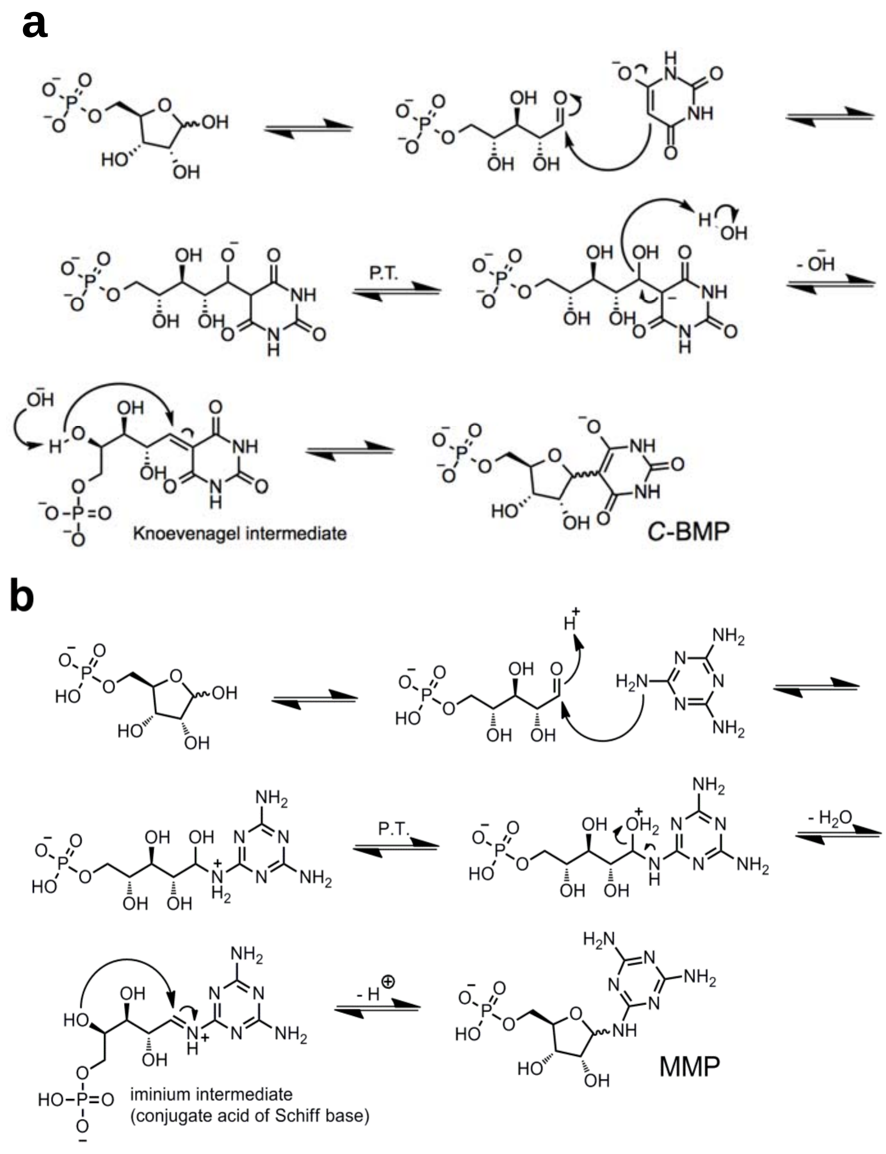

Supplementary Figure 8| Proposed mechanisms for nucleotide formation. a, Knoevenagel condensation results from attack of the nucleophilic C5 of deprotonated barbituric acid on the aldehyde of ribose-5-phosphate when the sugar is in its open form. Next, the C4′-OH group attacks the enone causing ring closure and formation of both anomers of the *C*-nucleoside. Note that the charge state of the molecules reflects reactions at pH from 7 to 9. **b**, A Schiff base is formed from attack of one of the exocyclic amine groups of melamine onto the aldehyde of ribose-5-phosphate followed by elimination of water. The iminium intermediate is attacked by the C4′-OH leading to ring closure and formation of both anomers of the exocyclic amino nucleotides. Note that the charge state of the molecules reflects the reaction at pH 5. P.T. indicates proton transfer.

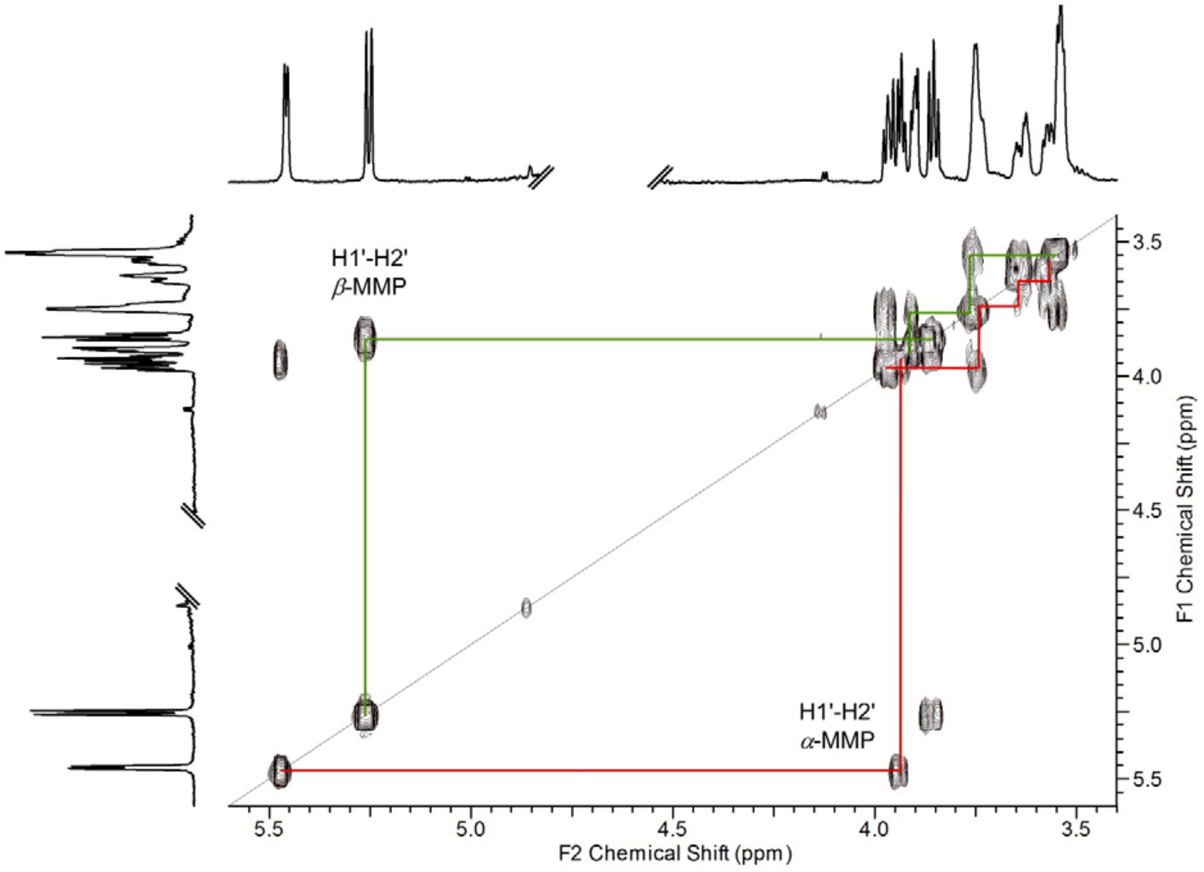

Supplementary Figure 9| ^{1}H and 2D COSY spectra of MMP. Along with HSQC and HMBC spectra present in Figure 3 of main text, COSY was used to confirm proton assignments of *α*-MMP and *β*-MMP. Red lines connect cross-peaks of *α*-MMP and green lines connect cross-peaks of *β*-MMP. The water peak was removed for clarity.

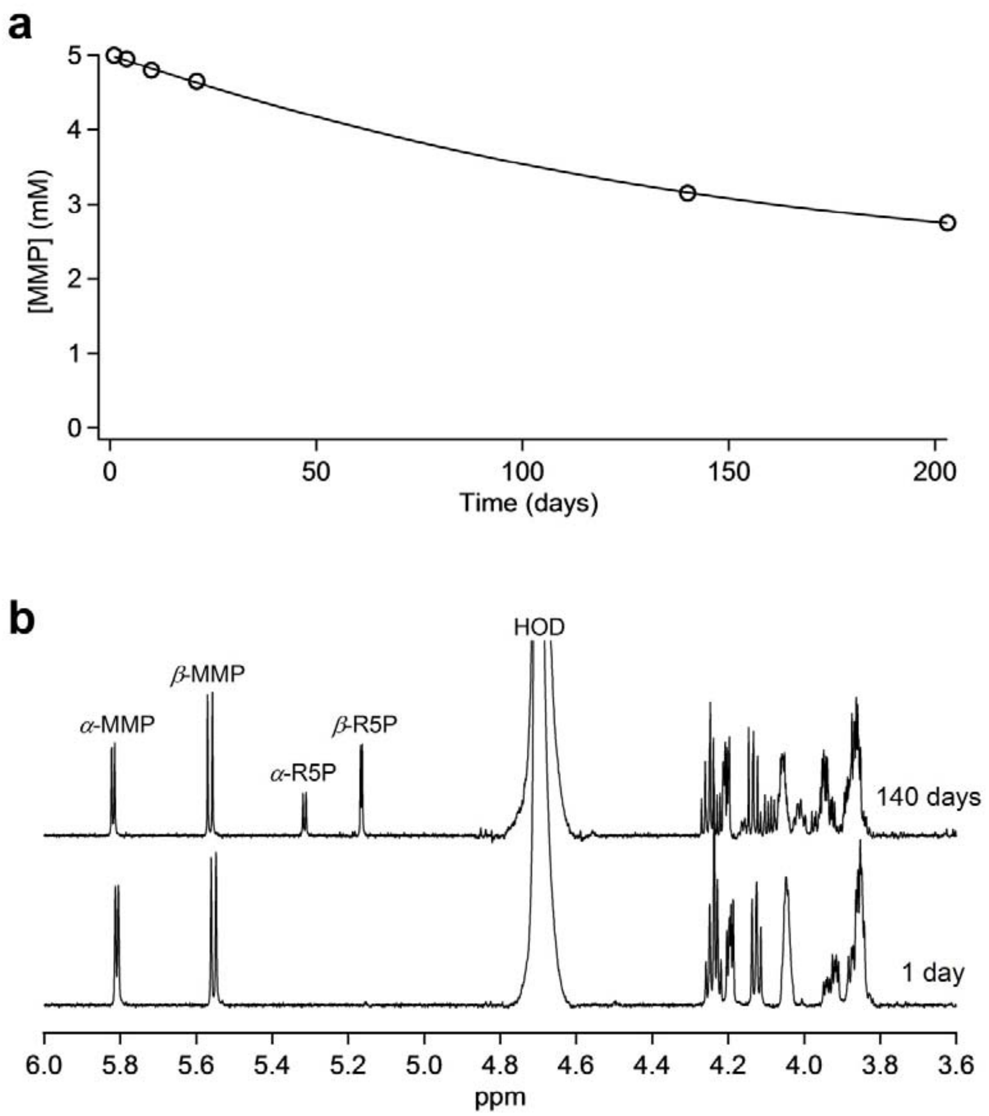

Supplementary Figure 10| Kinetics of MMP hydrolysis. a, Concentration of MMP as a function of time for a pH 5 aqueous solution originally 5 mM in MMP that was maintained at 5°C. Concentration of MMP was determined by measuring relative intensity of MMP anomeric proton resonances versus ribose anomeric proton resonances. Fit of data reveals a hydrolysis rate constant of 0.004 day^{-1} (ca. 6 month half-life) and dissociation constant (K_d) of 3.7 mM. **b**, Representative ^1H NMR spectra showing MMP solution after 1 and 140 days (anomeric protons of both anomers of MMP and R5P are labeled). The MMP solution also contained 300 mM NaCl. Equation used for fitting MMP concentration as a function of time in **a**: MMP(t) =

$$(K_d + 2*MMP0 - K_d^{1/2}*(K_d + 4*MMP0)^{1/2}*\tanh(((k_1*(K_d + 4*MMP0)^{1/2}*t)/K_d^{1/2} + 2*\mathrm{artanh}(K_d^{1/2}/(K_d + 4*MMP0)^{1/2}))/2))/2$$

Where MMP0 is the initial concentration of MMP.

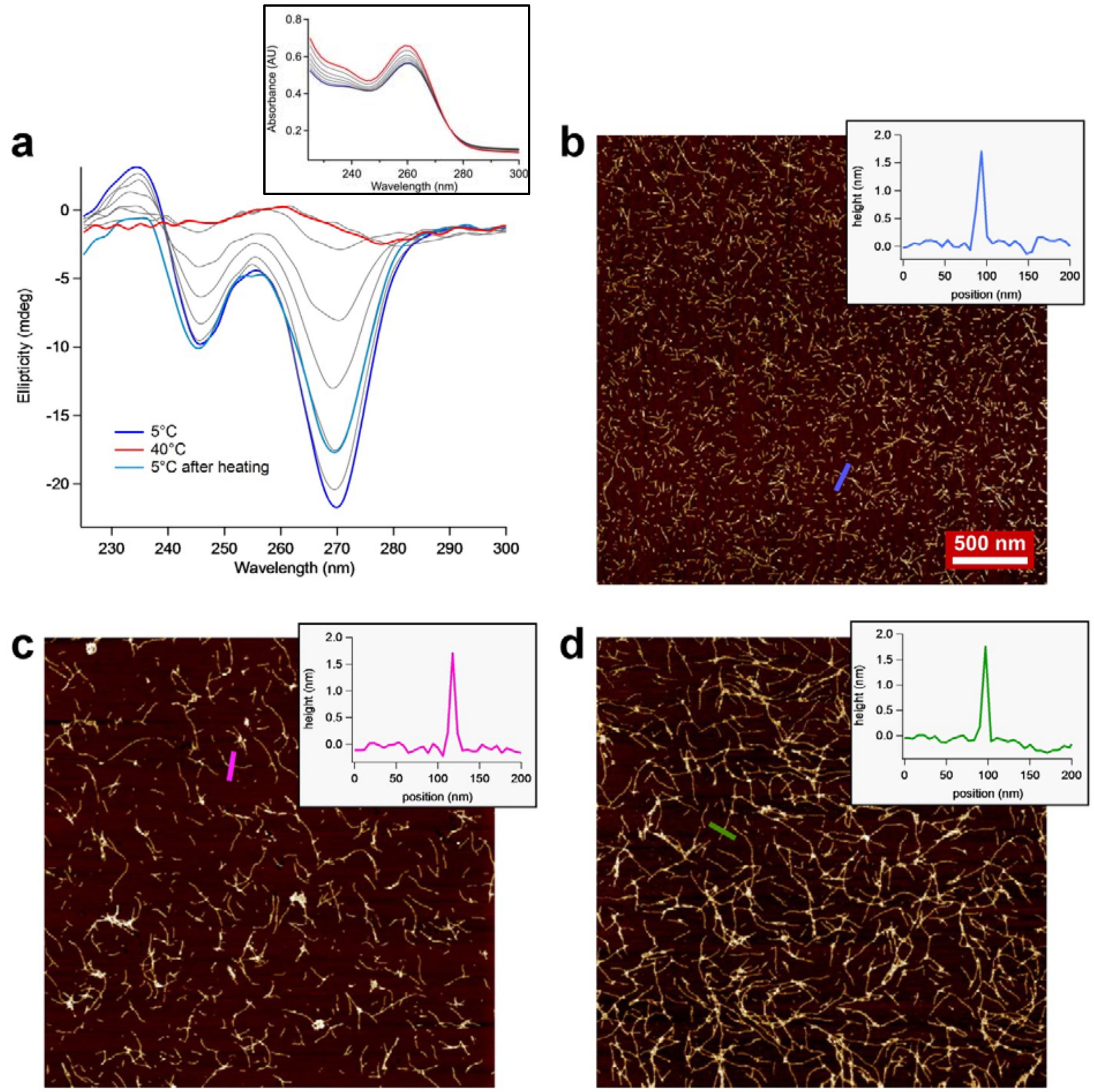

Supplementary Figure 11| CD and AFM analysis of supramolecular polymers containing MMP and/or *C*-BMP. a, CD spectra of a solution (50 mM in heterocycle of each solution of crude reaction products), ranging in temperatures from 5 to 40°C, and at 5°C after being heated twice to 40°C. Loss of signal when heated to 40°C and return of signal when cooled to 5°C illustrates the non-covalent nature of the assemblies formed in the mixture. Note that change in intensity with heat cycling is due to the kinetic behavior of supramolecular assembly nucleation and growth, which results in variations in the amount of chiral assembly formed for the same sample when cooled to 5°C after heating. Insert shows UV spectra of the same samples. (**b**-**d**) AFM topographic image of assemblies formed by combining **b**, purified MMP and purified *C*-BMP, **c**, purified *C*-BMP and melamine, and **d**, MMP and BA. Inserts show height profiles of fibers delineated by the colored line in the main panel. All solutions were 50 mM each monomer listed and contained 1 M NaCl.

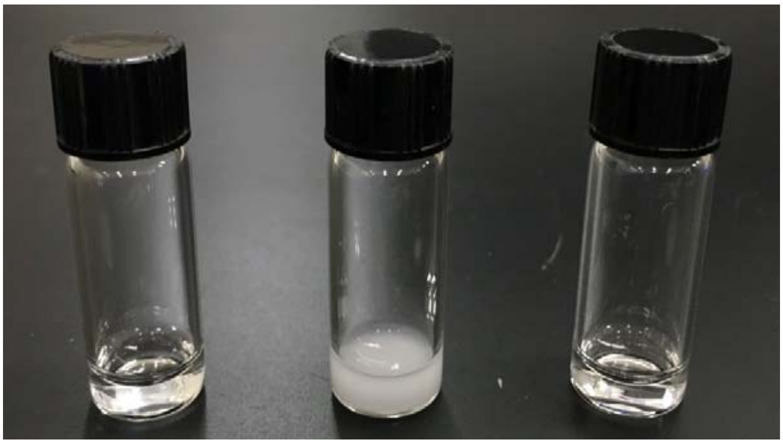

Supplementary Figure 12| Melamine and BA form an insoluble precipitate when mixed in water. Left vial contains 100 mM melamine, right vial contains 100 mM BA, and middle vial contains 50 mM melamine and 50 mM BA. Melamine and BA solutions were adjusted to pH 4.5 prior to mixing.

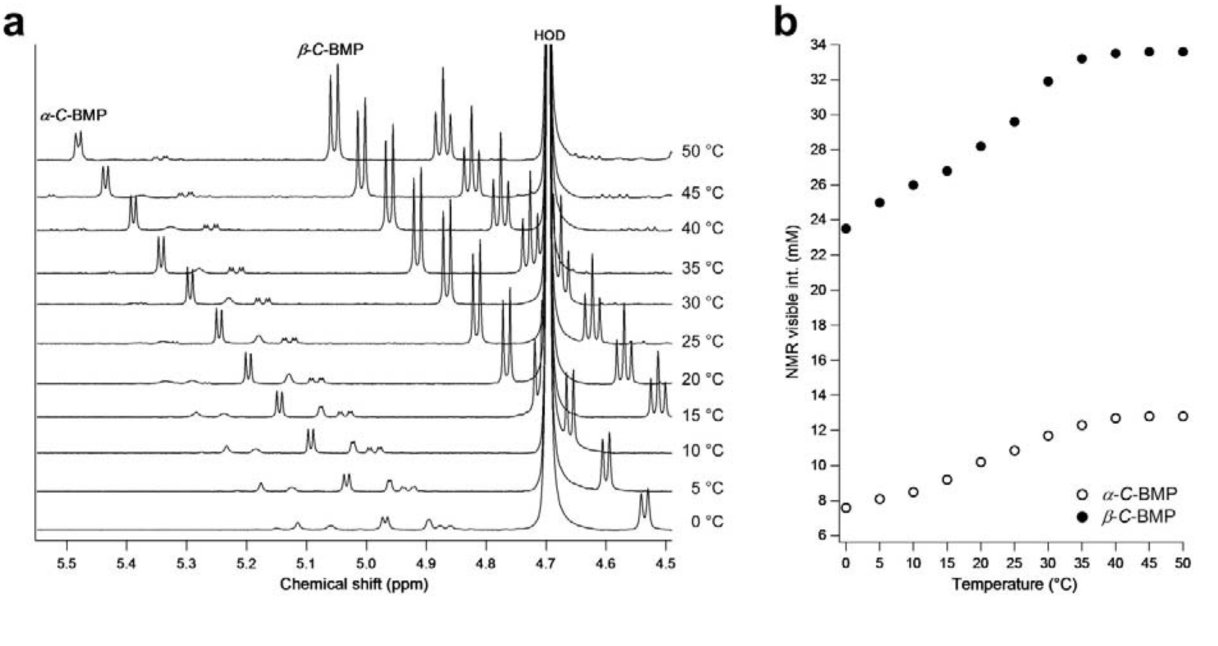

Supplementary Figure 13| VT-NMR of a solution containing *C*-BMP and melamine at 50 mM each. a, NMR spectra from 0 to 50°C showing temperature dependent assembly of both anomers of *C*-BMP with melamine. **b**, Concentration of *unassembled* *C*-BMP as a function of

temperature determined from the NMR visible intensities of both anomers. The HOD and anomeric proton resonances are labeled.

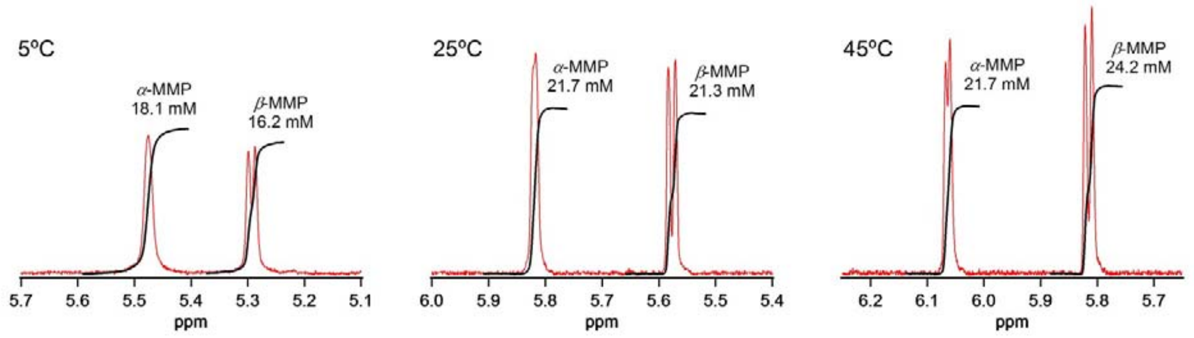

Supplementary Figure 14| VT-NMR of a solution containing MMP and BA at 50 mM each. Examples of anomeric proton regions of spectra used to determine the concentration of α-MMP and β-MMP in solution. All concentrations determined from 5 to 45°C are shown in Fig. 5a of main text. The solution contained 300 mM NaCl. Temperatures above 45°C were not evaluated because of an increase in anomerization at higher temperatures.
